# Hotspots and frontiers in PSMA research for prostate cancer: a bibliometric and visualization analysis over the past 20 years

**DOI:** 10.1186/s40001-023-01590-w

**Published:** 2023-12-19

**Authors:** Hanfei Zhang, Liu Xiao, Hangyu Xie, Lin Li

**Affiliations:** Department of Nuclear Medicine, West China Hospital, Sichuan University, No. 37, Guo Xue Xiang, Chengdu, 610041 China

**Keywords:** Bibliometric analysis, Prostate specific membrane antigen, Prostate cancer, VOSviewer, Citespace

## Abstract

**Background:**

Prostate-specific membrane antigen (PSMA)-targeted imaging and therapy have significantly changed the management of patients with prostate cancer (PCa) at different disease stages. This advancement has attracted the attention of scholars, leading to a prolific output of scholarly publications. This study comprehensively outlines the knowledge framework associated with PSMA-based diagnosis and treatment of PCa through the application of bibliometric analysis, and discusses the potential research trends and foci.

**Methods:**

Articles and reviews related to PSMA for prostate cancer from 2003 to 2022 were retrieved from Web of Science Core Collection. VOSviewer, Citespace, and R-bibliometrix were primarily employed to execute and visually represent co-authorship, co-citation, and co-occurrence analysis of countries, institutions, authors, references and keywords in this field.

**Results:**

A total of 3830 papers were included. The papers on the field of PSMA-based PCa therapy and imaging had been continuously increased since 2003, but the rate has slowed from 2020. The United States made the largest contribution in this field, in terms of publications 997 (26.03%), H-index (110) and total citations (53,167 times). We identified the most productive institution were Technical University of Munich, and Australian institutions had become very active in recent years. Journal of Nuclear Medicine was the most prominent journal in this field. Professors Matthias Eiber and Martin G Pomper made great achievements, while Ali Afshar-Oromieh was the most co-cited author. According to the result of keywords and topics analysis, “ga-68 labeled psma ligand”, “radiation dosimetry” and “HBED-CC” were major research areas in the near future, while "Extended pelvic lymph node dissection" was considered to be the future research foci.

**Conclusions:**

The field of psma-based PCa therapy and imaging is in the stage of vigorous development and has a bright prospect. The United States and Germany have achieved outstanding results in this area, while Australia has recently developed rapidly. It is foreseeable that more research foci will be lied in the early detection of pelvic lymph nodes and the multimodal imaging-guided surgery.

**Supplementary Information:**

The online version contains supplementary material available at 10.1186/s40001-023-01590-w.

## Introduction

Prostate cancer (PCa) is a significant contributor to tumor-related deaths in men, and as the population ages, more and more men are being diagnosed with PCa [1, 2]. Treatment choices for early localized PCa span from radical prostatectomy to external radiotherapy. In the case of advanced or metastatic PCa, androgen deprivation therapy is the preferred approach. However, it is noteworthy that the majority of tumors will ultimately progress into castration-resistant prostate cancer (CRPC) or metastatic castration-resistant prostate cancer (mCRPC). The 5-year survival rate of PCa range from 100% in early-stage tumors to as low as 30% in patients with advanced cancer [3]. This pressing need for improving the overall outcomes for patients with advanced cancer underscores the demand for novel therapies and early detection.

The Prostate-specific membrane antigen (PSMA) expression increases by 100 to 1000-fold in about 95% of PCa cells, making it an efficient target for imaging and treatment, particularly in aggressive types characterized by high Gleason scores (GS), as well as in cases of androgen-deprived, metastatic, and hormone-refractory PCa [4, 5]. The PSMA-ligand complex exhibits a distinctive behavior, being internalized through clathrin-coated pits and accumulating within endosomes [6]. This phenomenon contributes significantly to enhanced retention, which proves to be pivotal for optimizing both image quality and therapeutic effectiveness [7].

In recent years, radiotracers designed to target the PSMA for positron emission tomography (PET) have witnessed a growing utilization in cases of biochemical recurrence. These radiotracers have demonstrated their capability to identify disease sites that would remain concealed when employing conventional imaging modalities [8]. Besides, noteworthy advancements have been achieved in the management of mCRPC through innovative modalities like targeted radioligand therapy (RLT). RLT utilizes cytotoxic, highly energetic radioactive nuclides conjugated with high-affinity ligands, which specifically target overexpressed markers in cancerous tissue, leading to selective tumor removal while preserving healthy tissue [9, 10]. Additionally, theragnostics offer a promising avenue in nuclear medicine, referring to compounds that serve both therapeutic and imaging purposes [11]. Radiopharmaceuticals like PSMA617 exemplify the theragnostics concept as they can bind with positron-emitters (e.g., ^68^Ga, ^44^Sc, or ^152^Tb) for imaging and with alpha (α) ^225^Ac-emitters or beta minus (β-) ^177^Lu for treating previously identified tumor lesions [12–14]. This innovative approach facilitates disease imaging and targeted treatment, showcasing significant potential in the fight against PCa.

Considering the aspects described above, the investigation of PSMA in PCa has increasingly captivated researchers. However, the exponential proliferation of publications in this domain has rendered it progressively challenging for many researchers to stay abreast of the most recent research findings. Bibliometric analysis, as a convenient and novel method that allows quantitative and qualitative review of publications, successfully addressed this gap [15, 16]. Leveraging this method, researchers can swiftly delve into the contributions of authors and institutions, topic's evolution, main research domains, and promising directions within a specific research area [17].

This study utilized bibliometric analysis of publications indexed in the Web of Science Core Collection (WoSCC) database to achieve the following objectives: (i) illustrate the research trend of PSMA in PCa over the past decade; (ii) make an overall knowledge structure of this domain; (iii) identify and elucidate the challenging issues and research hotspots concerning PSMA in PCa; and (iv) offer valuable insights to guide future research endeavors in this area.

## Materials and methods

### Data source

**Fig. 1:**
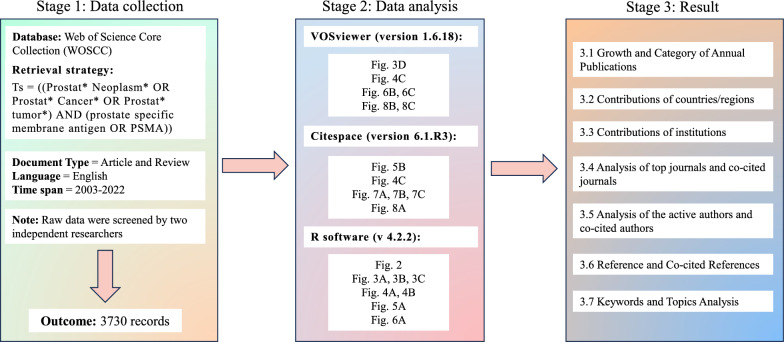
Flowchart of the publication selection in the study

In this work, the article data of PSMA in PCa were retrieved from Web of Science Core Collection (WOSCC) on 11 May 2023. For a better retrieval accuracy, a search strategy based on the standardized Medical Subject Headings (MeSH) list in the National Library of Medicine was applied. Two independent researchers performed the search strategy: Ts = ((Prostat* Neoplasm* OR Prostat* Cancer* OR Prostat* tumor*) AND (prostate specific membrane antigen OR PSMA)), with the timespan set from 2003 to 2022. In the current work, “articles” were selected as the final sample data for a depth analysis, since they are the most important document types as carriers of scientific knowledge. Besides, the types of language were restricted to English. Finally, 3830 publications were downloaded as “Plain text” with the record content “full record and cited references” (Fig. 1).

### Bibliometric analysis and visualization

Bibliometric analysis is a method performed to obtain high-level insights of the structure and patterns in academic publications. By applying mathematics and statistical methods to quantitative metrics representing informational about a research domain, scope, contents, and trends can be readily identified and visualized [18]. With the advent of the data science age, various scientometric analysis theories and visualization tools have been developed.

VOSviewer (version 1.6.18) is one of the most widely used tools, which provided serval functions for bibliometric mapping including collaboration analysis, topics analysis, and citations-based analysis [19]. In this work, VOSviewer was utilized to conduct author co-citation analysis, co-authorship analysis of countries/regions, authors, institutions, and topic analysis.

Citespace (version 6.1.R3) is another freely available citation visualization analysis software developed by Chen et al. [20, 21]. Compared with VOSviewer, it focuses on intellectual turning points and pivotal points in the development of a field [16]. In our research, we mainly adopted Citespace to accomplish co-citation analysis of references, dual-map overlay of journals, timeline diagram, and citation burst of keywords and references.

R (v 4.2.2)-Bibliometrix was used to analysis the growth and category of annual publications, visualize the cooperation network between countries, make the descriptive analysis of the publishing characteristics of journals and generate the distribution map of authors' annual publication volume over time. In addition, we used R software to create polar bar charts and radar charts to visualize the counts, total link strength, and total citations of institutions.

## Results

### Growth and category of annual publications

**Fig. 2:**
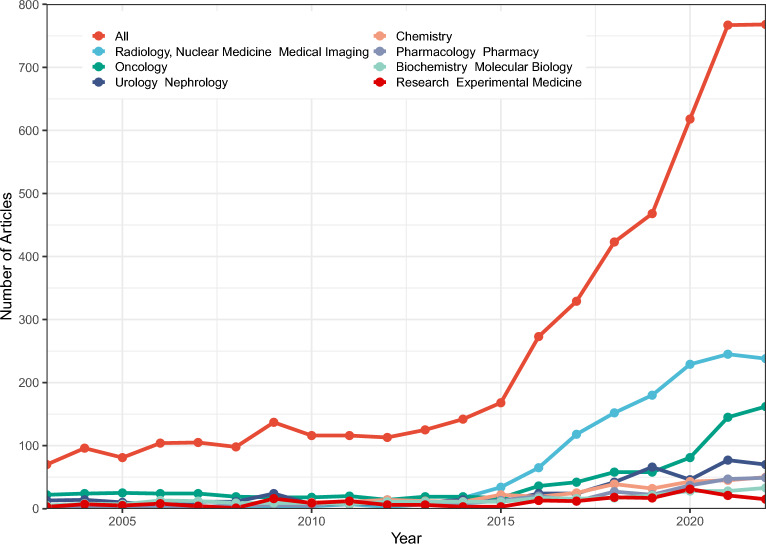
Global trend of publication on PSMA research for prostate cancer over the past 20 years

The publication trend in terms of the number of research papers demonstrates the scientific attention and popularity in a particular domain. In general, since 2003, the annual output of the PSMA research on PCa showed a progressive increase to the present (Fig. 2). Interestingly, we observed a “S-shaped” curve in publications growth. From 2003 to 2015, the annual publications have been increased gradually, but after 2015, the number of publications began to grow exponentially, suddenly jumping to 168 in that year, and reaching 767 papers in 2021. More recently, output in this area stayed relatively stable again.

Nearly half of the publications belonged to the radiology, nuclear medicine medical imaging and oncology category. Following categories were urology nephrology, chemistry, pharmacology pharmacy, and biochemistry molecular biology, etc. Noteworthy, the annual publications in radiology, nuclear medicine medical imaging increased before 2020, and then stayed stable, while these in oncology sustained growth, which indicated a lag in the category compared to radiology, nuclear medicine medical imaging.

### Contributions of countries/regions

**Fig. 3:**
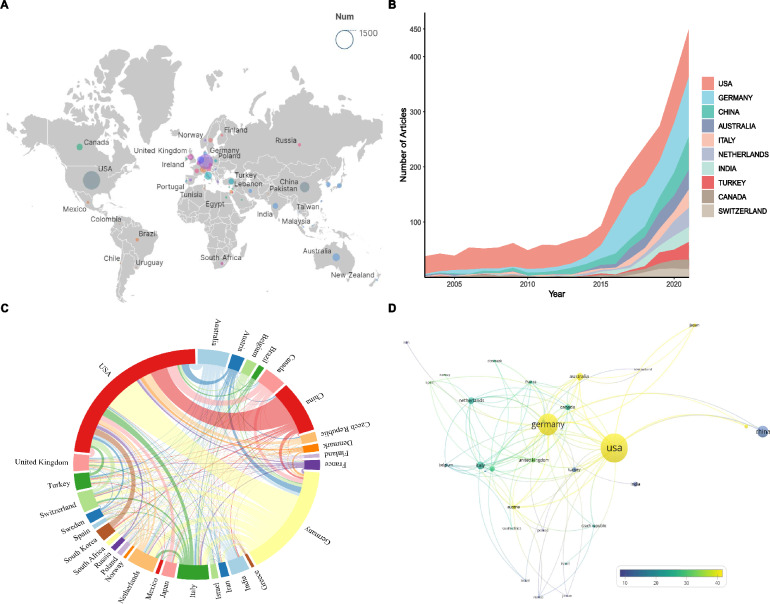
**A** Geographic distribution map based on the total publications of different countries/regions. **B** The changing trend of the annual publication quantity in the top 10 countries/regions over the past 20 years. **C** The cross-country/region collaborations visualization map. The thickness of the line between countries reflects the frequency of the cooperation. **D** The visualization map of countries/regions average article citations generated by using VOSviewer.

A total of 76 countries/regions had contributed in the field of PSMA in PCa. The world map based on cumulative publication number (Fig. 3A) indicated North America, Western Europe, and East Asia reached remarkable achievements in this field. Meanwhile, Fig. 3B showed the growing publication changes of the top 10 prolific countries from 2003 to 2022. As listed in Additional file 1: Table S1, the USA, and Germany ranked in the top two, with 997 (26.03%) and 658 (17.18%) publications, respectively. Moreover, H-index and total citations of the USA were also the highest among all the countries with 110 and 53,167 respectively, signifying that the USA was at the forefront of this field in the world.

Figure 3C showed the international collaboration on PSMA related research between countries/regions. The strongest collaborations links existed between Germany and the USA, and to a lesser degree, the USA and China. As shown in Fig. 3D a total of 30 countries were included and displayed. Among them, the top three countries with the highest average article citations were the USA (52.85), Germany (43.22), and Australia (37.14). Additionally, despite China's active participation in publishing numerous documents, the average citation count was relatively lower (12.73).

### Contributions of institutions

**Fig. 4:**
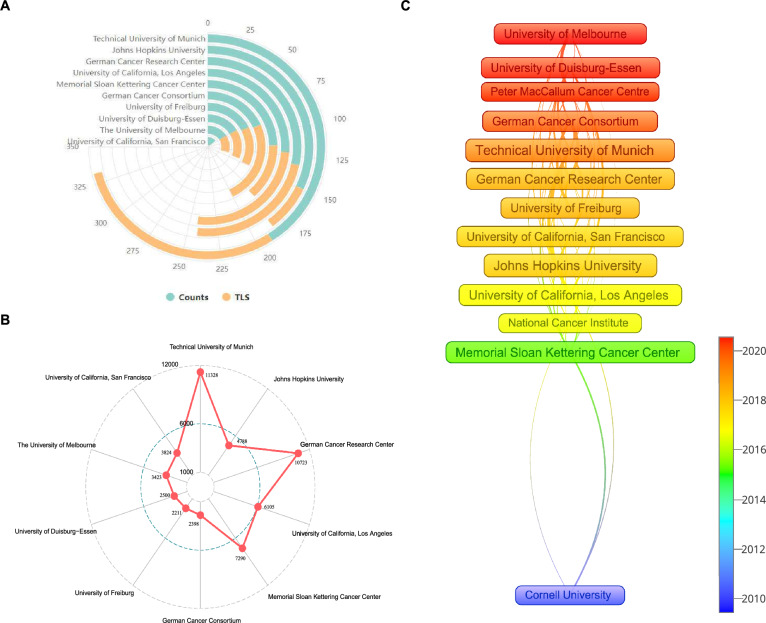
**A** The polar bar chart of counts, total link strength (TLS) of the top productive 10 institutions. **B** The radar bar chart of total citations of the top productive 10 institutions. **C** Average year of publication for the 13 most productive institutions

More than 1500 institutions participated in publishing research papers on PSMA in PCa. The polar bar chart in Fig. 4A summarized the counts and total link strength of the top productive 10 institutions in detail. It was evident that 9 institutions in the top 10 were from the USA and Germany, of which 5 were from Germany and 4 were from the USA. Specifically, Technical University of Munich ranked first with 190 papers, followed by Johns Hopkins University and German Cancer Research Center. Corresponding, the top three institutions with the largest total link strength (TLS) were Technical University of Munich (TLS = 142), University of California, Los Angeles (TLS = 121), and German Cancer Consortium (TLS = 114) (Additional file 1: Table S2).

As shown in Fig. 3B, the top three institutions with the highest total citations were Technical University of Munich (11328), German Cancer Research Center (10723) and Memorial Sloan Kettering Cancer Center (7290) (Additional file 1: Table S2).

The average publication year of active institutions were presented in Fig. 4C, visually indicated by the color evolution from dark blue to red. We selected 13 institutions, which published more than 60 research papers. Cornell University had temporally more distant publication years, indicating it was influential at earlier development stage, while University of Melbourne, University of Duisburg-Essen, and Peter MacCallum Cancer Centre had become very active in recent years.

### Analysis of top journals and co-cited journals

**Fig.5:**
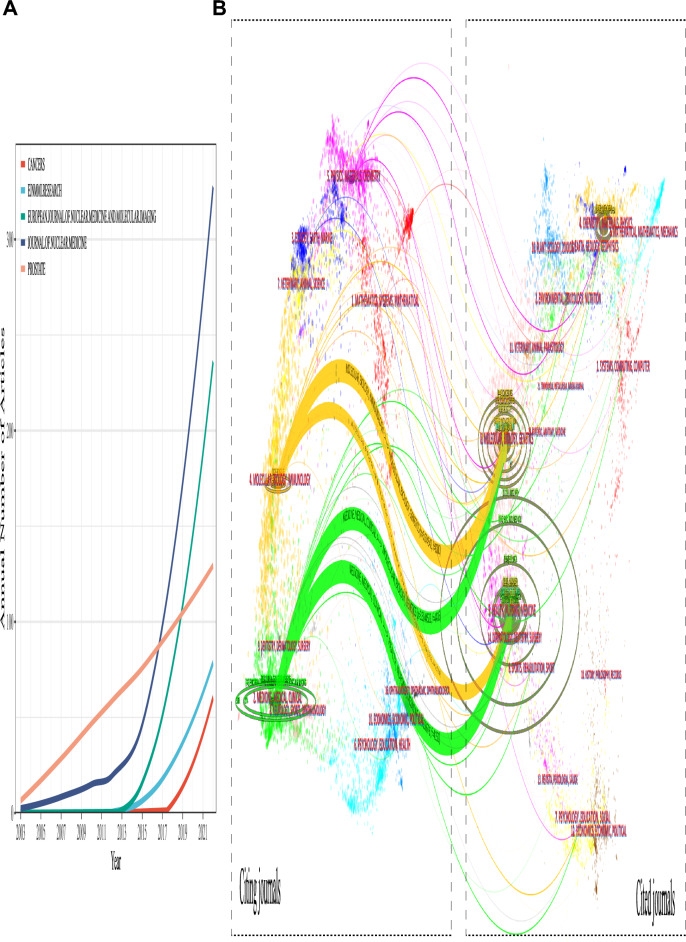
**A** Temporal analysis of publication sources for PSMA-related research on prostate cancer.** B** A dual-map overlap of journals with PSMA-related research on prostate cancer carried out by Citespace

At present, the articles had been published in 706 scholarly journals, with the annual number of publication sources increasing from 4 in 2005 to 412 in 2022. The top ten journals published 718 (42.3%) of the total number of papers, whereas 153 journals (53.1%) published only one paper in this domain. From the results of Table 1 and Fig. 5A, the most prolific journal is the *Journal of Nuclear Medicine*, with 318 articles, followed by *European Journal of Nuclear Medicine and Molecular Imaging* and *Prostate*.

**Table 1:** Top 10 Journals related to the research of PSMA-related prostate cancer

Rank	Sources	Counts	Total local citations	JCI^a^	IF^b^ (2021)	JCR^c^ quartile	Countries
1	Journal of Nuclear Medicine	318	12,085	2.58	11.082	Q1	USA
2	European Journal of Nuclear Medicine and Molecular Imaging	254	9288	2.32	10.057	Q1	Germany
3	Prostate	134	2926	1.08	4.012	Q3	USA
4	Cancers	83	404	1.03	6.575	Q1	Switzerland
5	EJNMMI research	79	1144	0.83	3.434	Q2	Germany
6	Clinical nuclear medicine	60	1748	0.97	10.782	Q1	USA
7	Frontiers in oncology	59	395	0.89	5.738	Q2	Switzerland
8	BJU international	53	1382	1.63	5.969	Q1	England
9	Nuclear medicine communications	53	520	0.41	1.698	Q4	USA
10	Clinical cancer research	51	3828	2.52	13.801	Q1	USA

^a^*JCI* Journal Citation Indicator, ^b^I*F* Impact Factor, ^c^ *JCR* Journal Citation Reports

A dual-map overlay was an analytical approach, which was used to represent clusters of involved journals focusing on similar themes. As rendered in the Fig. 5B, the left clusters represented research frontier or hotspot, while the right clusters indicated where they cited. It could be found that the literature published in *‘Molecular, Biology, Genetics’* and *‘Health, nursing, medicine’* cluster was frequently used together as a basis for advancing PSMA research associated with the *‘Medicine, medical, clinical’* journal clusters. Besides, the clusters ‘*Molecular, Biology, Genetics’* and ‘*Health, nursing, medicine’* are commonly found together to advance ‘*Molecular, Biology, Immunology*’ related PSMA research.

### Analysis of the active authors and co‑cited authors

Table 2 summarized the top 10 authors with the largest number of documents and top 10 co-cited authors. Matthias Eiber was the most productive researcher, followed by Martin Pomper and Uwe Haberkorn. Among the top ten most productive researchers, eight were from Germany and two were from the USA.

**Table 2:** The 10 most productive authors and top 10 co-cited authors in PSMA-related prostate cancer research

Rank	Author	Country	Documents	Total Citations	TLS^a^	Co-Cited Author	Country	Total Citations	TLS^a^
1	Matthias Eiber	Germany	138	9032	510	Ali Afshar-Oromieh	Germany	2090	20,923
2	Martin G Pomper	USA	136	5304	221	Matthias Eiber	Germany	973	9977
3	Uwe Haberkorn	Germany	80	8691	274	Michael S Hofman	Australia	937	8251
4	Klaus Kopka	Germany	70	6314	202	Wolfgang P Fendler	Germany	922	9654
5	Steven P Rowe	USA	70	2141	212	Clemens Kratochwil	Germany	836	8476
6	Hans-Jürgen Wester	Germany	70	5745	170	Kambiz Rahbar	Germany	762	8438
7	Ken Herrmann	Germany	67	4032	261	Sam S. Chang	USA	670	5311
8	Tobias Maurer	Germany	67	5514	204	Matthias Eder	Germany	656	7117
9	Clemens Kratochwil	Germany	54	5455	196	Silver DA	USA	647	6251
10	Wolfgang P Fendler	Germany	50	3508	212	Tobias Maurer	Germany	640	6394

^a^TLS: Total link strength

**Fig. 6:**
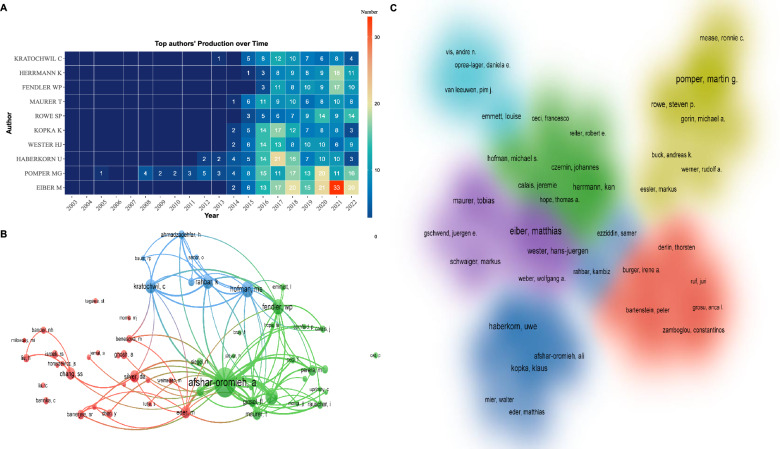
**A** The distribution map of the top 10 most prolific authors over time generated by R software. **B** Author co-citation analysis by VOSviewer. One node represents an author, and the lines between nodes represent the co-citation relationship. **C** Author co-authorship analysis by VOSviewer. One color reprents a cluster, and authors with close relationship are allocated to the same cluster

Figure 6A depicted the annual outputs of these top 10 authors between 2003 and 2022, where dark blue color corresponds to higher outputs while the yellow color corresponds to the lower outputs. Figure 6B was a visualization map of author co-citation analysis generated by VOSviewer. The node size in the map reflects the relationship of the author with other authors in the network. Through co-citation analysis, we found that Ali Afshar-Oromieh ranked first with 2090 total citations, which is more than twice as many as Matthias Eiber (973 times), indicating that he was at the center of the research in this field. Figure 6C showed the cluster density map of author co-authorship analysis. Only 50 authors with more than 26 papers were included, forming a total of 6 author clusters.

## Reference and co-cited references

**Fig. 7:**
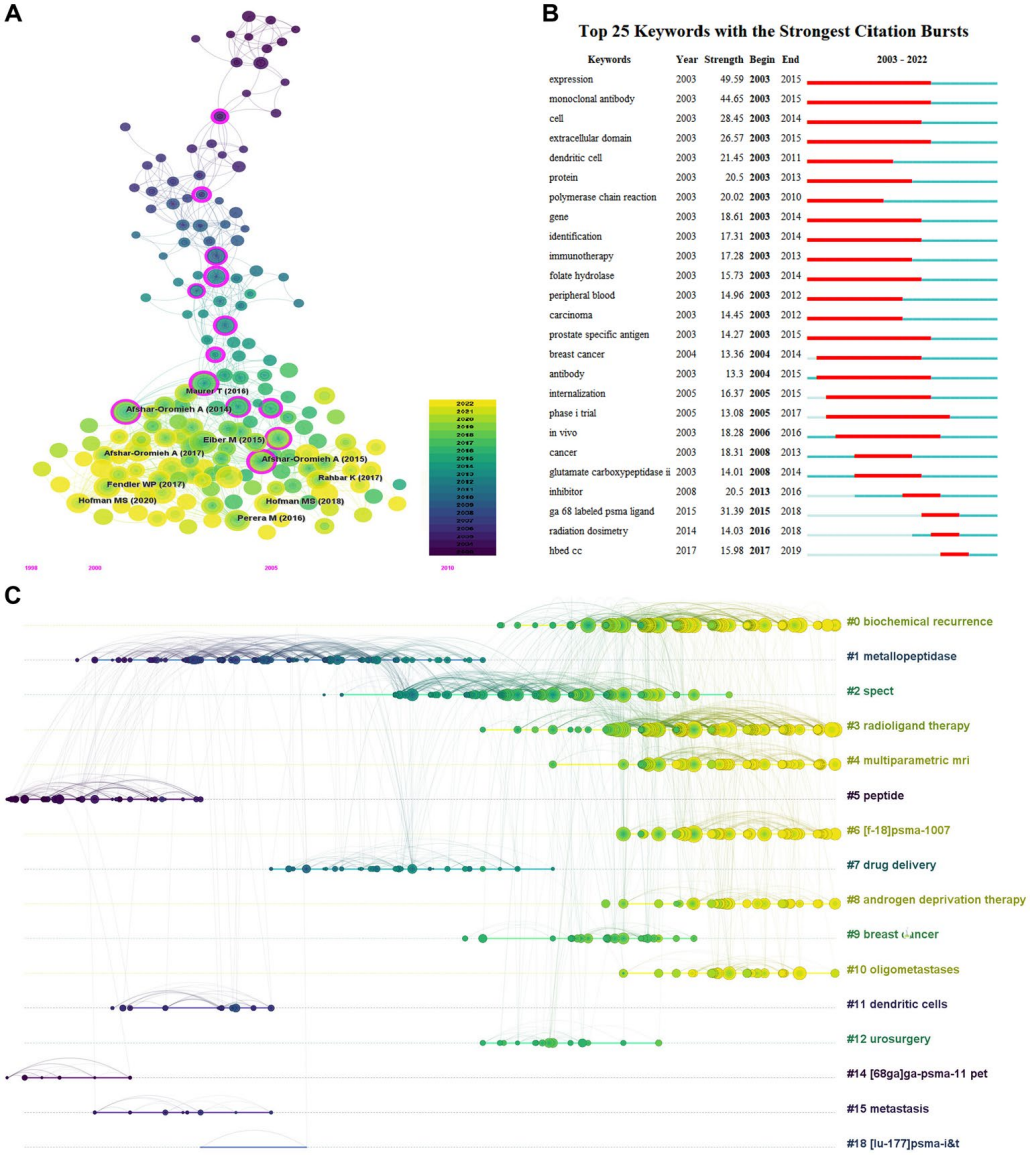
**A** The visualization map of reference co-citation analysis produced by Citespcae. A node with a high betweenness centrality is shown with a purple ring. **B** The visualization map of top 25 references with the strongest citation bursts in PSMA-related prostate cancer research. **C** A network map displaying co-citation references in a timeline view. The time evolution is indicated with different colored lines and the nodes on the lines indicate the references cited

We listed the top ten local cited papers in Table 3. The most cited article was published by Matthias Eiber in 2015 [22], with a total of 436 times, followed by Ali Afshar-Oromieh [23] and Ali Afshar-Oromieh (2013) [24], with 383 and 361 times respectively. Co-cited references refer to a set of references that are frequently cited together in scholarly documents. According to Fig. 7A, the three most frequently co-cited references were Matthias Eiber [22], Marlon Perera [25], and Ali Afshar-Oromieh [26] with citation counts of 346, 286, and 284 respectively.

**Table 3:** Top 10 original articles concerning the research of PSMA-related prostate cancer

Article	First author	Journal	Total Local Citation	Total Global Citation	Publication Year
Evaluation of Hybrid ^68^Ga-PSMA Ligand PET/CT in 248 Patients with Biochemical Recurrence After Radical Prostatectomy	Matthias Eiber	J Nucl Med	436	776	2015
Comparison of PET imaging with a (68)Ga-labelled PSMA ligand and (18)F-choline-based PET/CT for the diagnosis of recurrent prostate cancer	Ali Afshar-Oromieh	Eur J Nucl Med Mol Imaging	383	778	2014
PET imaging with a [68 Ga]gallium-labelled PSMA ligand for the diagnosis of prostate cancer: biodistribution in humans and first evaluation of tumour lesions	Ali Afshar-Oromieh	Eur J Nucl Med Mol Imaging	361	649	2013
Tumor target prostate specific membrane antigen (PSMA) and its regulation in prostate cancer	Arundhati Ghosh	J Cell Biochem	350	569	2004
68 Ga-complex lipophilicity and the targeting property of a urea-based PSMA inhibitor for PET imaging	Matthias Eder	Bioconjug Chem	350	608	2012
The diagnostic value of PET/CT imaging with the (68)Ga-labelled PSMA ligand HBED-CC in the diagnosis of recurrent prostate cancer	Ali Afshar-Oromieh	Eur J Nucl Med Mol Imaging	348	600	2015
[177Lu]-PSMA-617 radionuclide treatment in patients with metastatic castration-resistant prostate cancer (LuPSMA trial): a single-centre, single-arm, phase 2 study	Michael S Hofman	Lancet Oncol	292	628	2018
68 Ga-PSMA PET/CT: Joint EANM and SNMMI procedure guideline for prostate cancer imaging: version 1.0	Wolfgang P Fendler	Eur J Nucl Med Mol Imaging	290	439	2017
Diagnostic Efficacy of (68)Gallium-PSMA Positron Emission Tomography Compared to Conventional Imaging for Lymph Node Staging of 130 Consecutive Patients with Intermediate to High Risk Prostate Cancer	Tobias Maurer	J Urol	285	531	2016
Prostate-specific membrane antigen PET-CT in patients with high-risk prostate cancer before curative-intent surgery or radiotherapy (proPSMA): a prospective, randomised, multicentre study	Michael S Hofman	Lancet	275	686	2020

In Fig. 7B, the map illustrated the top 25 papers with the most significant citation bursts. The blue line represented the time interval, while the red line indicated the duration of the citation burst, revealing the evolution of research hotspots over time. The most recent reference citation burst was identified in 2020 and has persisted until the present. Notably, among them, the paper on urea-based PSMA inhibitor for PET imaging authored by Matthias Eder et al. in 2012 exhibited the highest strength value.

As shown in Fig. 7C, the references were categorized into 18 clusters, each exhibiting active co-citation relationships. The timeline view of co-citation references is a visual diagram that can gain insights into the evolution of research trends. Currently, the research focus on biochemical recurrence (#0), radioligand therapy (#3), multiparametric MRI (#4), ^18^F-PSMA-1007 (#6), androgen deprivation therapy (#8), and oligometastases (#10), indicating a growing interest among researchers in these areas of study.

## Keywords and topics analysis

**Fig. 8:**
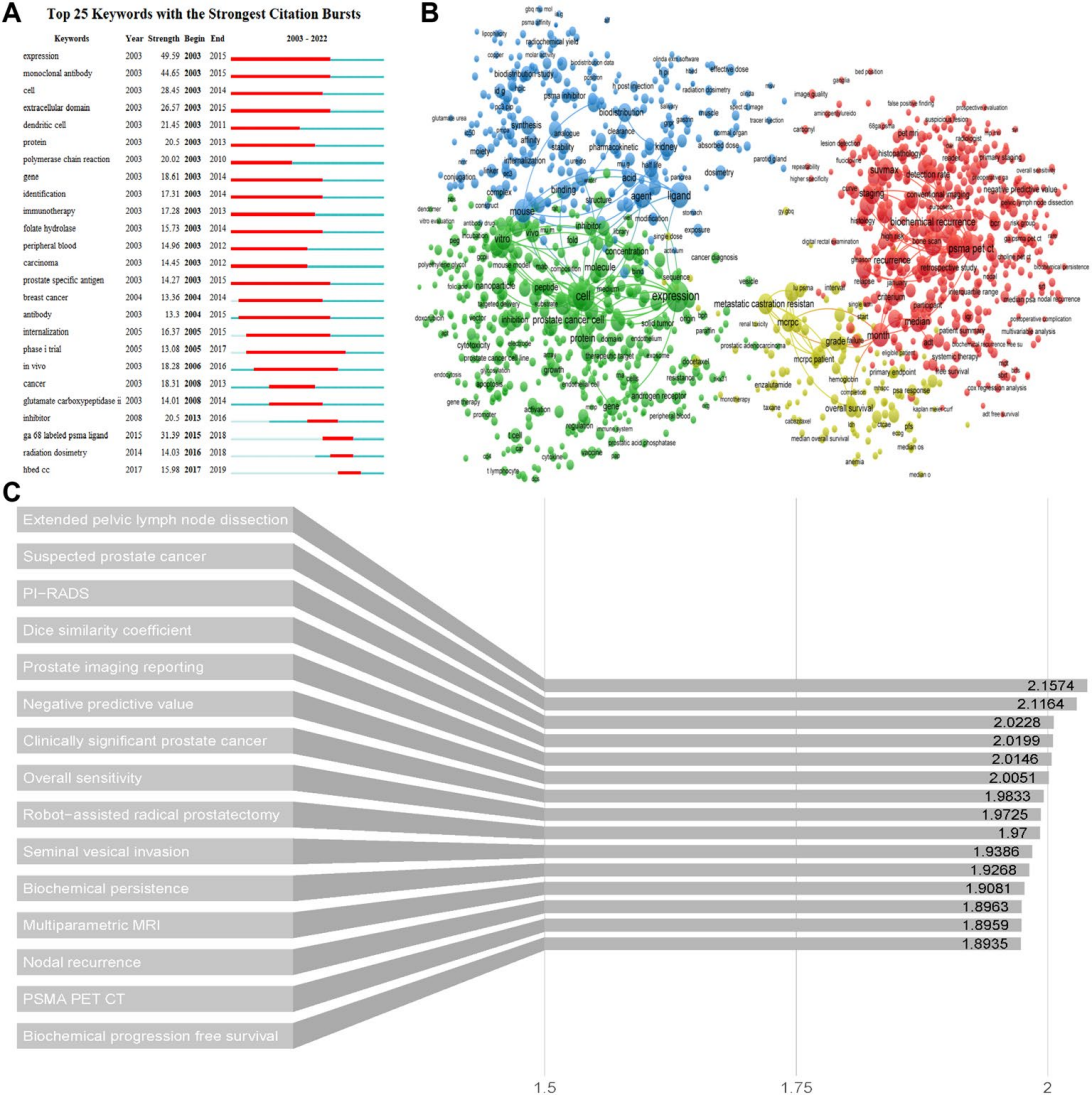
**A** Visualization map of top 25 keywords with the strongest citation bursts in PSMA-related prostate cancer research. **B** The overlay visualization map of author terms co-occurrence analysis. **C** The top 15 terms with the highest relevance score

Figure 8A presented a map of the top 25 keywords exhibiting strong citation bursts by Citespace. The prominent keywords, including “HBED-CC”, “radiation dosimetry”, and "^68^Ga labeled PSMA ligand," indicated significant research interest in these areas in recent years. However, none of the keywords kept bursting to the present.

To analyze the primary terms and research trends within the PSMA domain, we employed text mining and visualization techniques to extract and formulate a distribution map from the titles and abstracts of the entire collection of 3830 papers by VOSviewer. PSMA itself, such as 'Prostate specific membrane antigen' and 'antigen' were excluded. Only subjects that were present in a minimum of 10 documents are taken into account, resulting in 1390 topics that surpass this threshold.

As shown in Fig. 8B, four sub-domains of PSMA research were clustered together and distinguished by different colors. Cluster #1 tumor detection and medical equipments (red); Cluster #2 PSMA expression (green); Cluster #3 biodistribution, drug synthesis and animal models (blue); Cluster #4 target population and radioligand therapy (yellow). Additional file 1: Fig. S1 clearly reflected the evolution of PSMA research topics in recent years, from clustering related to cellular experiments, such as "Expression" and "Tumor Cells," to specific applications, such as "PSMA PET CT" and "Radioligand Therapy" (Additional file 1). Figure 8C summarized the top 15 terms with the highest relevance score.

## Discussion

In the context of the information explosion in the scientific realm, staying attuned to research hotspots and keeping abreast of the latest research findings pose significant challenges, emphasizing the importance of bibliography retrieval and knowledge management as essential tasks for researchers. Distinct from systematic reviews or meta-analyses, bibliometric analysis stands out as a valuable approach, enabling the comprehensive summarization of the development of specific research fields and the analysis of research hotspots. As an innovative and pioneering study, this research utilizes bibliometric methods to provide a comprehensive overview of the application and development of PSMA in PCa research over the past 20 years.

To a certain extent, the number of scientific articles reflect the development of research in a particular field. In general, publications on PSMA in the field of PCa exhibited exponential growth until 2020, but it has recently entered a plateau phase. The annual publication trend in the category of radiology, nuclear medicine medical imaging is consistent with this, but the number of annual publications in the oncology category continues to grow steadily. A lag between the two categories suggested although PSMA has become a star molecule in the field of nuclear medicine, increasing evidence suggests the high sensitivity of PSMA-targeted imaging and the effectiveness of PSMA RLT for PCa. However, the manner and timing of its application in tumor patients are currently undergoing intensive testing within the oncology field [27–29]. The prosperity in publications and research in the field of nuclear medicine is poised to provide a deeper understanding of the mechanisms behind PSMA-targeted therapy and imaging, thereby potentially providing widespread benefits to all PCa patients.

Based on the findings of the country/regional distribution, the USA (997, 26.03%) held the highest count of published articles, followed by Germany (658, 17.18%) and China (343, 8.96%) within the 76 countries/regions involved in this study. Together, these three countries accounted for 52.17% of the entire paper output, signifying their prominence in PSMA-related PCa research. However, China's overall citation was unsatisfactory, notably its average citations per paper, which ranked third lowest among the top 10 countries/regions in terms of research productivity (Additional file 1: Table S1). This reveals an enduring imbalance of scholarly resources between developing and developed nations. Developing countries such as China, India, and Turkey, while being prolific in publication output, still exhibited a dearth of highly-cited and high-quality research, resulting in limited global influence.

Typically, cooperating countries tend to be geographically interconnected and revolve around the leading country in terms of publishing productivity [30]. The USA serves as the principal leader in international collaborations, maintaining close partnerships with Germany, China, and Canada. Our study manifested that most cooperation and research communication in the field of PSMA were limited to North America, Europe and a few Asian countries. Consequently, future prospects underscore the significance of international cross-border collaborations, particularly with developing regions and countries. Besides, China, as an Asian country, is following the trend of globalization and is actively participating in cooperative research in this field.

The Technical University of Munich emerged as the foremost institution in terms of productivity and influence, maintaining robust collaborative ties with numerous countries/regions. Nevertheless, while certain Chinese institutions, such as Shanghai Jiao Tong University and Nanjing Medical University, demonstrated prolific paper publication and noteworthy academic impact, substantial international academic partnerships and exchanges with institutions from other countries remained relatively limited (Additional file 1: Fig. S2). From Fig. 4C, it can be observed that institutions in Australia, such as the University of Melbourne and the Peter MacCallum Cancer Centre, engaged in PSMA research for PCa at a later stage compared to institutions in the United States and Germany. This observation indicated that Australian institutions have recently started becoming active in this field, suggesting the potential for them to gain a more significant position in the future.

Identification of significant journals can offer researchers a valuable reservoir of dependable reference information, aiding them in selecting appropriate target journals for literature search and research submissions [31]. The analysis revealed that the top 10 journals spanned across quartiles Q1 to Q4, with impact factors (IF) ranging from 1.698 to 13.801, signifying the varying article quality in the realm of PSMA-related studies in PCa. According to Bradford’s Law, the ten most influential journals were determined, including *Journal of Nuclear Medicine*, *European Journal of Nuclear Medicine and Molecular Imaging*, Prostate, *Cancers*, *EJNMML Research* and *Clinical Nuclear Medicine* (Additional file 1: Fig. S3). These journals played a pivotal role in PSMA research during the study period. Notably, the *Journal of Nuclear Medicine* and *European Journal of Nuclear Medicine and Molecular Imaging* were essential contributors in nuclear medicine, indicating that PSMA was one of the hotspots in nuclide-based PCa therapy.

In author co-authorship analysis, the top 10 most active authors are all from the USA and Germany, and they have published more than 800 papers. Among them, Martin G Pomper entered this research field at an earlier stage, and his first publication can be traced back to 2005 [32]. In this publication, his team synthesized and evaluated two radiolabeled urea derivatives for their in vivo biodistribution, demonstrating the feasibility of PSMA-based imaging agents for PCa imaging. Moreover, Martin G Pomper and Steven P Rowe are colleagues in Johns Hopkins University, and they have been engaged in collaborative research for a long period time, producing a large amount of literature related to molecular imaging [33–35]. In 2012, Matthias Eiber, Uwe Haberkorn, Tobias Maurer, and Ali Afshar-Oromieh et al. pioneered the utilization of hybrid 68 Ga-PSMA ligand PET/CT for evaluating patients with biochemical recurrence after radical prostatectomy. They established a comprehensive database and published numerous key papers on this subject [26, 36–38]. This work played a pivotal role in shaping the landscape of nuclear medicine and molecular imaging. In the context of PCa research focused on PSMA, we believe significant contributions might emerge from the above team members. Enhancing collaboration with these prominent teams is a good choice for research.

Citation analysis and co-citation analysis of references are significant methods within bibliometric studies. In our study, we used total local citation to assess the impact and influence of a particular publication within PSMA research domain. Specifically, the articles by Matthias Eiber et al., “Evaluation of Hybrid ^68^Ga-PSMA Ligand PET/CT in 248 Patients with Biochemical Recurrence After Radical Prostatectomy” published on *Journal of Nuclear Medicine* had been cited 436 times, which was the most cited article in this field. His team found that 68 Ga-PSMA ligand PET/CT was more effective than CT in identifying lymph node metastases, local recurrence, or bone metastases [22]. The second most local cited article was published in 2014 by Ali Afshar-Oromieh. The study found that 68 Ga-PSMA PET/CT detected significantly more lesions characteristic for PCa when compared to choline-based PET/CT. Additionally, 68 Ga can be extracted from a commercially available 68Ge/68 Ga radionuclide generator without the need for cost-intensive cyclotrons [23].

Burst detection is an algorithmic technique devised to capture rapid surges in the popularity of references or keywords during a specific period, offering an effective approach to detect hotspots or interest. Our results indicated that the initial burst of reference citations in this domain started in 2005 and has persisted until the present. It was due to the research on the Phase I trial of 177Lu-J591 in patients with androgen-independent PCa, which demonstrated that the radiopharmaceutical was well-tolerated and had biologic activity. Figure 7C showed that two important references are still frequently cited. One was published by Wolfgang P Fendler, his research team demonstrated 68 Ga-PSMA-11 PET imaging has high positive predictive value, detection rate, and inter-reader agreement for localization of recurrent prostate cancer [39]. The other study published by Michael S Hofman concluded that [^177^Lu]-PSMA-617 treatment has high response rates, low toxic effects, and reduces pain in patients with metastatic castration-resistant prostate cancer who have progressed after conventional treatments [40].

Keywords burst detection in Fig. 8C generated from Citespace showed that the first detected keyword was “expression” in 2003, which was also had the strongest strength value. It is the high level of PSMA expression up to 1000-fold in PCa epithelial secretory cells that makes it a target for diagnostic and therapeutic isotope labeling. Given the increasing utilization of PSMA-based imaging agents, a substantial number of extraprostatic tissues manifesting elevated PSMA uptake have been detected, especially triple-negative breast cancer, providing novel prospective applications for PSMA radioligand therapy and imaging [41–44]. From 2015 to 2019, “ga-68 labeled psma ligand”, “radiation dosimetry” or “HBED-CC” had become popular researches areas. ^68^ Ga-PSMA-11 (HBED-CC) is the first ^68^ Ga radiopharmaceutical approved by the U.S. Food and Drug Administration (FDA) in 2020, and understanding the human dosimetry data for these radiopharmaceuticals is critical for clinical applications [45, 46].

Co-occurrence networks for extracting terms from titles and abstracts of publications is a widely used method in bibliometrics to identify popular research topics. VOSviewer computes a relevance score for each noun phrase. Generally, noun phrases with lower relevance scores exhibit broader concepts, while those with higher scores usually denote more specific meanings [47]. As shown in Fig. 8C, these terms include cutting-edge PSMA diagnostic and therapeutic conditions and evaluation criteria applicable to PCa, with 'Extended pelvic lymph node dissection (ePLND)' having the highest relevance. EPLND is currently the mainstay of lymph node staging for newly diagnosed PCa [48, 49]; however, this overtreatment is of particular concern due to the prolonged duration of the procedure and the risk of ePLND-related complications [50–52]. Therefore, there exists a pressing necessity for innovative imaging modalities to effectively refine the indications for ePLND. In this context, PSMA PET/CT has demonstrated superior accuracy than traditional imaging methodologies in both pelvic and distant assessment [53, 54]. The literature suggested that the high negative predictive value (NPV) observed in individuals with diminished risk of lymph node invasion have important utility in mitigating the incidence of unwarranted ePLND [55]. In addition, recent investigations have demonstrated that the radioisotopic tagging of PSMA ligands using gamma-emitting radionuclides like 99 m-technetium can be employed for the execution of PSMA-based radio-guided surgery (PSMA-RGS) [56]. This method, in theory, holds the potential to enhance the localization of nodal metastases during surgical procedures and enable the detection of micrometastatic lesions that might escape detection through preoperative conventional and molecular imaging, primarily owing to their inherent limitations in spatial resolution [57, 58]. Many studies have also reported multimodal imaging-guided surgery, which is a integration of conventional imaging modalities, PSMA-based radiologic guidance, PSMA-based fluorescence guidance, etc. [59, 60]. If intraoperative guidance is introduced during the initial stages of radical prostatectomy, it has the potential to significantly extend the time before tumor metastasis or recurrence occurs. Furthermore, it holds promise for achieving a definitive cure, representing the future direction of research endeavors.

## Limitations

There are some limitations worth noting in this study. First, the restriction to articles published in English can lead to certain biases and blind spots in the analysis, as there are likely also a significant number of articles in other major languages, such as Chinese, Spanish, and Hindi [61]; Secondly, this study only included the publications from 2003 to 2022, and it was possible to miss some important and landmark studies before 2002. Finally, due to the continuous updating of database, recently published high-quality articles may be underestimated for their unsatisfactory citations [31].

## Conclusions

In summary, our results show that PSMA-targeted examination and treatment for PCa are being adopted at an unprecedented rate and have made great progress, indicating that precision treatment of PCa is expected to be achieved in the future. To date, the USA continues to dominate the field of PSMA-based PCa research, followed by Germany, both of which play an important role in driving further development of the field. In addition, Australian institutions have become notably active in this field recently, with the potential to bring greater opportunities to this domain. The key foci of PSMA-based PCa research in the future lie in the early detection of pelvic lymph nodes and the development of the multimodal imaging-guided surgery. Unfortunately, at present, the growth of PSMA related research papers in PCa field is slowing down, possibly because many PSMA-targeting studies are still in the preclinical research stage. What we need is to generate reliable patient outcome data at different clinical stages to better understand their clinically relevant indications and improve the diagnosis and treatment of PCa patients.

### Supplementary Information

**Additional file 1: Figure S1.** Average publication year of terms by VOSviewer. **Figure S2.** The institutional cooperation map created with Citespace. **Figure S3.** The core resource classified by Bradford Law generated by R software. **Table S1.** Top 10 productive countries/regions in PSMA-related prostate cancer research. **Table S2.** The 10 most productive institutions in PSMA-related prostate cancer research.

## Data Availability

The datasets supporting the conclusions of this article were retrieved from using the Web of Science database.

## Abbreviations

PCa: Prostate cancer
CRPC: Castration-resistant prostate cancer
mCRPC: Metastatic castration-resistant prostate cancer
PSMA: Prostate-specific membrane antigen
PET: Positron emission tomography
RLT: Radioligand therapy
TLS: Total link strength
ePLND: Extended pelvic lymph node dissection
NPV: Negative predictive value
FDA: Food and Drug Administration
PSMA-RGS: PSMA-based radioguided surgery
