# Supplementary material for: Hotspots and frontiers in PSMA research for prostate cancer: a bibliometric and visualization analysis over the past 20 years
Source: Eur J Med Res. 2023 Dec 19;28:610. doi: 10.1186/s40001-023-01590-w (PMC10731714; doi:10.1186/s40001-023-01590-w)
Supplement: Supplementary file 1 — Additional file 1: Figure S1. Average publication year of terms by VOSviewer. Figure S2. The institutional cooperation map created with Citespace. Figure S3. The core resource classified by Bradford Law generated by R software. Table S1. Top 10 productive countries/regions in PSMA-related prostate cancer research. Table S2. The 10 most productive institutions in PSMA-related prostate cancer research. [file 40001_2023_1590_MOESM1_ESM.pdf]

Additional File

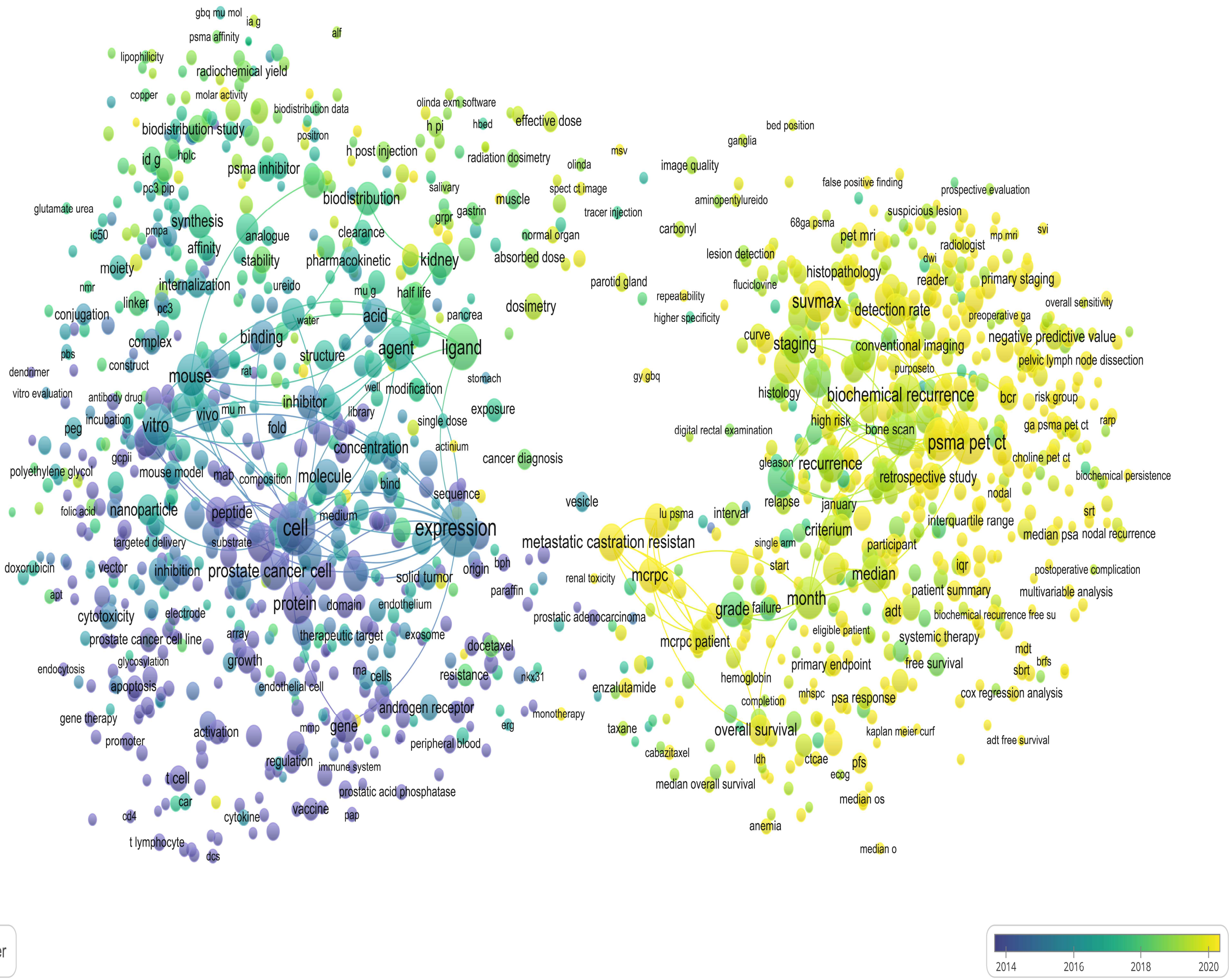

Additional file 1 Figure S1 Average publication year of terms by VOSviewer.

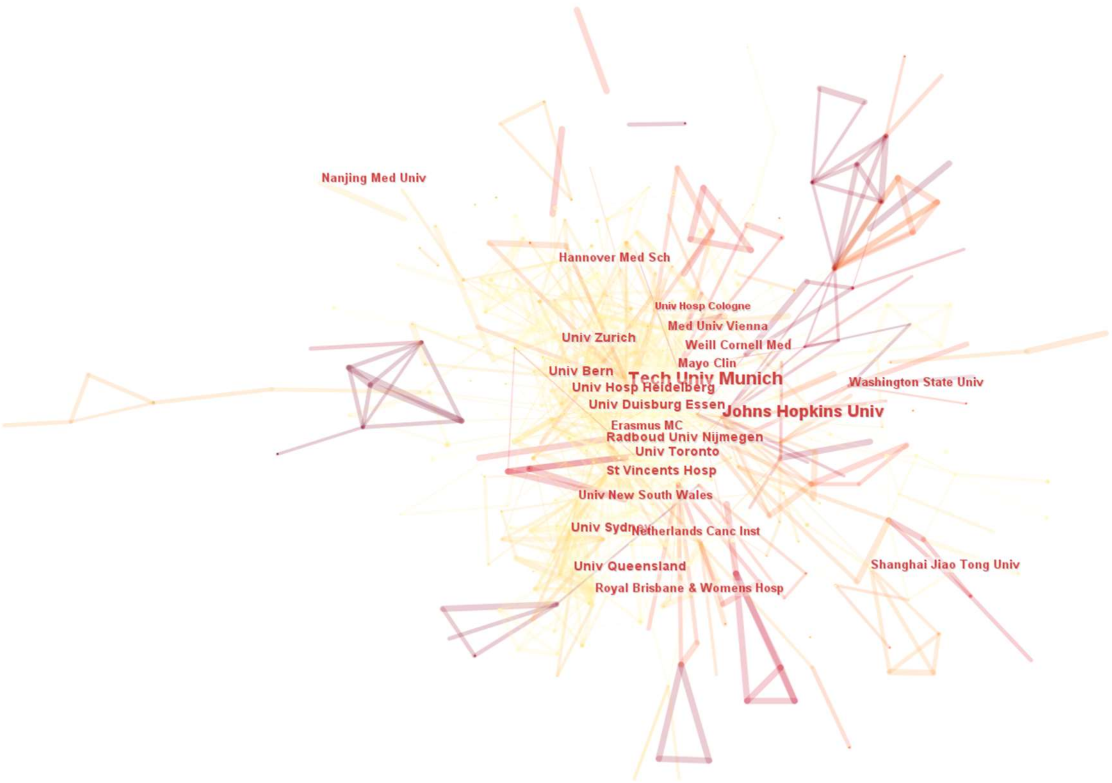

Additional file 1 Figure S2 The institutional cooperation map created with Citespace.

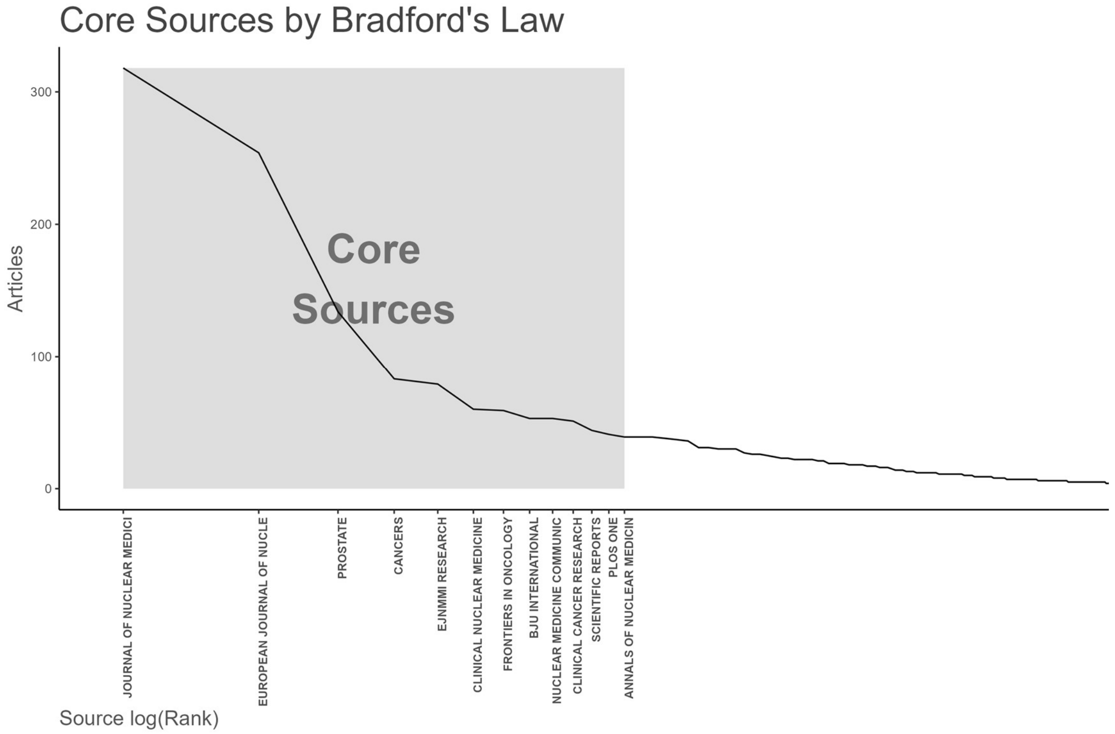

Additional file 1 Figure S3 The core resource classified by Bradford Law generated by R software.

Additional file 1 Table S1 Top 10 productive countries/regions in PSMA-related prostate cancer research

Rank	Country	Number	Percentage	Total Citations	Average Article Citations	H-index
1	USA	997	26.03%	53167	52.85	110
2	Germany	658	17.18%	29520	43.22	87
3	China	343	8.96%	4671	12.73	35
4	Australia	188	4.91%	7279	37.14	43
5	Italy	150	3.92%	2213	13.92	25
6	Netherlands	140	3.66%	2508	17.06	29
7	India	118	3.08%	1213	9.94	18
8	Turkey	112	2.92%	1185	10.39	18
9	Canada	97	2.53%	1966	18.55	25
10	Switzerland	83	2.17%	1898	22.07	26

Additional file 1 Table S2 The 10 most productive institutions in PSMA-related prostate cancer research

Rank	Organization	Documents	Citations	Total Link Strength	Country
1	Technical University of Munich	190	11328	142	Germany
2	Johns Hopkins University	149	4788	35	USA
3	German Cancer Research Center	132	10723	110	Germany
4	University of California, Los Angeles	122	6105	121	USA
5	Memorial Sloan Kettering Cancer Center	115	7290	64	USA
6	German Cancer Consortium	86	2398	114	Germany
7	University of Freiburg	82	2211	67	Germany
8	University of Duisburg-Essen	76	2500	92	Germany
9	The University of Melbourne	75	3423	68	Australia
10	University of California, San Francisco	67	3824	62	USA
